# Inhibition of nitric oxide and inflammatory cytokines in LPS-stimulated murine macrophages by resveratrol, a potent proteasome inhibitor

**DOI:** 10.1186/1476-511X-11-76

**Published:** 2012-07-10

**Authors:** Asaf A Qureshi, Xiu Qin Guan, Julia C Reis, Christopher J Papasian, Sandra Jabre, David C Morrison, Nilofer Qureshi

**Affiliations:** 1 Department of Basic Medical Sciences, School of Medicine, University of Missouri, 2411 Holmes Street, Kansas City, MO, 64108, USA; 2 Division of Pharmacology and Toxicology, School of Pharmacy, University of Missouri-Kansas City, 2464 Charlotte Street, Kansas City, MO, 64108, USA; 3 Department of Microbiology and Immunology, University of Maryland, 685 W. Redwood Street, Baltimore, MD, 21201, USA

**Keywords:** Nitric oxide (NO), TNF-α, NF-κB, Cytokines, Resveratrol, Proteasome inhibitors

## Abstract

**Background:**

Altered immune function during ageing results in increased production of nitric oxide (NO) and other inflammatory mediators. Recently, we have reported that NO production was inhibited by naturally-occurring proteasome inhibitors (quercetin, δ-tocotrienol, and riboflavin) in lipopolysaccharide (LPS)-stimulated RAW264.7 cells, and thioglycolate-elicited peritoneal macrophages from C57BL/6 mice. In a continuous effort to find more potent, non-toxic, commercially available, naturally-occurring proteasome inhibitors that suppress inflammation, the present study was carried out to describe the inhibition of NF-κB activation and NO, TNF-α, IL-6, IL-1β, and iNOS expression by trans-resveratrol, trans-pterostilbene, morin hydrate, and nicotinic acid in LPS-induced RAW 264.7 cells and thioglycolate-elicited peritoneal macrophages from C57BL/6 and BALB/c mice.

**Results:**

The present results indicate that resveratrol, pterostilbene, and morin hydrate caused significant inhibition (>70% to 90%; ***P*** < 0.02) in the activities of chymotrypsin-like, trypsin-like, and post-acidic (post-glutamase) proteasome sites in RAW 264.7 cells at a dose of only 20 μM. These compounds also inhibited the production of NO by RAW-264.7 cells stimulated with LPS alone (>40%; ***P*** < 0.05), or LPS + interferon-γ (IFN-γ; >60%; ***P*** < 0.02). Furthermore, resveratrol, pterostilbene, morin hydrate, and quercetin suppressed secretion of TNF-α (>40%; ***P*** < 0.05) in LPS-stimulated RAW 264.7 cells, and suppressed NF-κB activation (22% to 45%; ***P*** < 0.05) in LPS-stimulated HEK293T cells. These compounds also significantly suppressed LPS-induced expression of TNF-α, IL-1β, IL-6, and iNOS genes in RAW 264.7 cells, and also in thioglycolate-elicited peritoneal macrophages from C57BL/6 and BALB/c mice.

**Conclusions:**

The present results clearly demonstrate that resveratrol and pterostilbene are particularly potent proteasome inhibitors that suppress expression of genes, and production of inflammatory products in LPS-stimulated RAW 264.7 cells, and macrophages from C57BL/6 and BALB/c mice. Resveratrol and pterostilbene which are present in grapes, blueberries, and red wine, have been implicated as contributing factors to the lower incidence of cardiovascular disease in the French population, despite their relatively high dietary fat intake. Consequently, it appears likely that the beneficial nutritional effects of resveratrol and pterostilbene are due at least in part, to their ability to inhibit NF-κB activation by the proteasome, thereby suppressing activation of pro-inflammatory cytokines and iNOS genes, resulting in decreased secretion of TNF-α, IL-1β, IL-6, and NO levels, in response to inflammatory stimuli. This is the first report demonstrating that resveratrol and pterostilbene act as proteasome inhibitors, thus providing a mechanism for their anti-inflammatory effects.

## Background

Alterations in normal regulation of immune function occur during the ageing process, as well as in a variety of pathologic human conditions [1-3]. We have been particularly interested in dysregulation of immune function associated with ageing, as manifested by enhanced nitric oxide (NO) production by macrophages from senescent mice [4], and the potential impact of reversing these age-associated alterations in immune regulation. Toward this end, we reported that several naturally-occurring proteasome inhibitors (e.g. quercetin, δ-tocotrienol, riboflavin) inhibit production of inducible nitric oxide synthase (iNOS), nitric oxide (NO) and tumor necrosis factor-alpha (TNF-α) by lipopolysaccharide (LPS)-stimulated RAW 264.7 cells and murine macrophages in vitro, and that dietary supplementation with these compounds has comparable suppressive effects on the secretion of TNF-α and production of NO *in vivo* and ex vivo [5,6]. Our research has focused primarily on macrophages because they are highly sensitive to LPS stimulation and respond by producing TNF-α, interleukin-1β (IL-1β), IL-6, IL-8, NO, NF-κB, and activator protein-1 (AP-1), which are largely responsible for many of the pathophysiological events associated with gram-negative sepsis and other inflammatory diseases [5-7].

**Figure 1:**
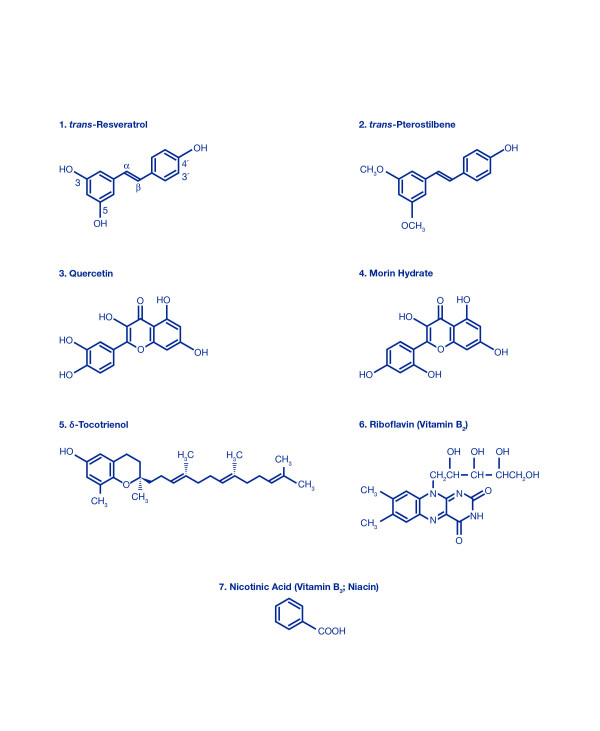
Chemical structures of various compounds used in this study.

The present study was carried out to identify additional non-toxic, commercially available, naturally-occurring proteasome inhibitors with potent anti-inflammatory properties. After initially screening a large number of compounds for their effect on proteasome protease activities, we opted to study trans-resveratrol, trans-pterostilbene, morin hydrate, and nicotinic acid (niacin; vitamin B_3_), more thoroughly; quercetin was included as a positive control, because we have carefully characterized this compound in previous publications [5-7]. Resveratrol, pterostilbene, morin hydrate, nicotinic acid (niacin) and quercetin are commonly found in nature, and have antioxidant, free-radical scavenging, anti-inflammatory, and hypolipidemic properties [8-13]. Pterostilbene is a methoxy ester of meta-phenolic (hydroxyl) groups of resveratrol, and morin hydrate contains meta-hydroxy groups compared to ortho-hydroxy groups present in quercetin (Figure 1). The positive physiological effects of resveratrol, pterostilbene, morin hydrate, nicotinic acid (niacin; vitamin B_3_), and quercetin have been reported in the literature and these agents have been approved by the FDA for human consumption for many years [8-14]. Resveratrol, pterostilbene and quercetin are active components in grapes, blueberries and red wine, contributing to the lower incidence of cardiovascular disease in the French population [8,9], and morin hydrate (isoflavonoid found in tea leaves) has been shown to be an effective hypocholesterolemic agent [12].

We carried out the present investigation to specifically demonstrate the relative capacity of these compounds (trans-resveratrol, trans-pterostilbene, morin hydrate, nicotinic acid, and quercetin) to inhibit key proteasomal enzymatic activities (e.g. chymotrypsin-like, trypsin-like, and post-glutamase activity), and production of NO, the iNOS enzyme, and certain pro-inflammatory cytokines (TNF-α, IL-1β, and IL-6). In order to make sure that the effectiveness of these compounds remains uniform and consistently reproducible their anti-inflammatory properties were examined in LPS-stimulated RAW 264.7 cells and in thioglycolate-elicited peritoneal macrophages prepared from 8-week-old female C57BL/6 and BALB/c mice.

## Materials and methods

### Reagents

Highly purified, deep rough chemotype LPS (Re-LPS) from *E. coli* D31m4 was prepared as described [15]. Dulbecco’s Modified Eagle Medium (DMEM) heat-inactivated low-endotoxin fetal bovine serum (FBS) and gentamicin were purchased from Cambrex Proteasome-Glo reagents and luciferase assay system for determining the proteasomal activities (Walkersville, MD, USA) for tissue culture studies. were purchased from Promega (Madison, WI, USA). Thioglycolate was purchased from Sigma, Aldrich Chemical Co. (St. Louis, MO, USA) and RNeasy mini kit from QIAGEN Sciences (Germantown, MD, USA). The murine macrophage cell line, RAW 264.7 cells (ATCC #. TIB-71) was purchased from American Type Culture Collection (Manassas, VA, USA), and HEK293T cells (ATCC #. CRL-11268; human embryonic kidney cells) was purchased from American Type Culture Collection (Rockville, MD, USA). *trans*-Resveratrol was purchased from “Mega Resveratrol” (60 Newtown Toad # 32, Danbury CT, USA), *trans*-pterostilbene from Shanxi Yong Yuan, Biotechnology Co, Ltd. (China), and nicotinic acid (niacin, vitamin B_3_) from VOIGT Global Distribution Inc. (P.O.Box. 1130, Lawrence, Kansas, USA). (−) Riboflavin and morin hydrate were purchased from Sigma-Aldrich Chemical Co (St. Louis, MO, USA). Quercetin was purchased from Alfa Aesar (Johnson Matthey Co. Lancastor, UK). SuperFect transfection reagent (Flag-TLR4 wild type + pELAM-luciferase {NF-κB reporter} + β-gal reporter plasmids + MD-2 + CD14 constructs) was purchased from QIAGEN, Valencia, CA, USA. Luciferase assay lysis buffer, and Luciferase assay system were obtained from Promega, Madison, WI, USA. β-Galactosidase was purchased from Tropix; Galacto Light System from Bedford, MA, USA. Berthold LB 9507 Luminometer was purchased from Berthold Technologies, Oak Ridge, TN, USA, and Stratagene staining kit from Stratagene, La Jolla, CA, USA. The 50 % δ-tocotrienol fraction from annatto seeds was purchased from American River (Boston, MA, USA), and purified from that fraction by flash chromatography as described previously [7]. The purity of δ-tocotrienol was established by high pressure liquid chromatography (HPLC) against its standard as reported earlier [7]. *trans*-Resveratrol and *trans*-pterostilbene will be referred to as resveratrol and pterostilbene, respectively, throughout this manuscript.

### Animals

6-week-old female, C57BL/6, Wild Type (WT) and BALB/c mice were obtained from the Jackson Laboratory (Bar Harbor, ME, USA). Mice used in this study received humane care in compliance with the principles of laboratory animal care formulated by the National Society of Health Guide for the “Care and Use of Laboratory Animals” by the US National Society of Health (NIH Publication No 85–23, revised 1996). The experimental procedures involving animals were reviewed and approved by the “Institutional Animal Care and Use Committee of UMKC”, Medical School, MO. All 6-week-old mice were acclimatized to the new environment for 14 days before beginning experimentation. Mice were fed *ad libitum* regular commercial mouse diet and had free access to water throughout the experiment. A 12 h light and 12 h dark cycle was maintained throughout the study.

### Cell culture and maintenance

RAW 264.7 cells or thioglycolate-elicited peritoneal macrophages derived from 8-week-old C57BL/6 or BALB/c mice were cultured in DMEM supplemented with 10% heat inactivated FBS and 10 mg/mL gentamicin at 37°C in a humidified, 5% CO_2_/95% air atmosphere as described previously [5,16,17].

### Effects of resveratrol, pterostilbene, morin hydrate, nicotinic acid, and quercetin on proteasomal chymotrypsin-like, trypsin-like, and post-glutamase enzymatic activities in RAW 264.7 whole cells

The comparative inhibitory effects of resveratrol, pterostilbene, morin hydrate, nicotinic acid, and quercetin on the chymotrypsin-like, trypsin-like, and post-glutamase activities of proteasomes in cultured RAW 264.7 whole cells were carried out essentially as reported recently [5]. Briefly RAW 264.7 cells (1x10^4^ cells/well) were allowed to adhere to wells in white plates (96-well, Fisher, 0877126) for 2 h, followed by the addition of various concentrations of resveratrol, pterostilbene, morin hydrate, nicotinic acid, or quercetin [10, 20, 40, 80, 160, or 320 μM in 100 μL; dissolved in 0.2 % dimethyl sulfoxide (DMSO)]. The mixtures were incubated at 37°C in an incubator in a 5% CO_2_/95% air atmosphere, for 60 min, and allowed to equilibrate to room temperature for 20 minutes before adding the Proteasome-Glo reagent (Promega, Madison, WI, USA). Proteasome-Glo reagent (100 μL) was added to each well to a total volume of 200 μL/well (tris buffer, pH 7.5; 0.02 M). The plates were covered with a plate sealer and kept in the dark at room temperature for 30 min. The relative luminescence units (RLU) of assays were read in a Promega Plate Luminometer. The chymotrypsin-like, trypsin-like, or post-glutamase activities were quantified by measuring luminescence after treatment of RAW 264.7 whole cells with various concentrations of each compound in a Luminometer (Promega, Madison, WI, USA), according to the manufacturer’s directions.

### Effects of resveratrol, pterostilbene, morin hydrate, nicotinic acid (niacin), and quercetin on the production of nitric oxide (NO) by RAW 264.7 cells stimulated with medium only, LPS, or LPS + interferon-γ (IFN-γ)

RAW 264.7 cells (1x10^6^ cells/500 μL/well) were allowed to adhere to tissue culture wells for 2 h. After 2 h, cells were treated with medium, resveratrol, pterostilbene, morin hydrate, nicotinic acid, or quercetin (16 μM of each compound in 100 μL medium; dissolved in 0.2 % DMSO) for 60 min (pretreatment). Cells were then challenged with LPS (10 ng/well; 400 μL) alone, LPS + IFN-γ (10 ng + 50 U/well) or medium alone and incubated in an incubator at 37°C in a humidified, 5% CO_2_/95% air atmosphere, for 36 h. After incubation, the supernatants were collected and stored at −20°C. Levels of NO were determined by measuring the amount of nitrite, a stable metabolic product of nitric oxide, as previously reported [17]. The assay mixture contained medium (100 μL) plus Griess reagent (100μL), and absorption was measured at 570 nm using a “Microplate Reader” (MR 5000; Dynatech Laboratories, Inc. Chantilly, VA, USA). The amount of nitrite was determined by comparison of unknowns using a NaNO_2_ standard curve. The NO detection limit was 0.20 nM [18].

### Effects of resveratrol, pterostilbene, morin hydrate, nicotinic acid, and quercetin on the secretion of TNF-α by LPS-stimulated RAW 264.7 cells

RAW 264.7 cells (1x10^6^ cells/500 μL/well) were allowed to adhere to tissue culture wells for 2 h. After 2 h, cells were treated with resveratrol, pterostilbene, morin hydrate, nicotinic acid, or quercetin (16 μM of each compound in 100 μL medium; dissolved in 0.2 % DMSO) for 60 min (pretreatment). Cells were then challenged with LPS (10 ng/well; 400 μL) or medium alone and incubated at 37°C in a humidified, 5% CO_2_/95% air atmosphere for 4 h. After 4 h incubation, supernatants were collected and stored at -20°C. The levels of TNF-α in supernatants were measured by Quantikine M ELISA kit (R&D System, Minneapolis, MN, USA) according to the manufacturer’s instructions [5-7]. The lower limit of detection for TNF-α by this method is approximately, 5.0 pg/mL [5].

### Effects of resveratrol, pterostilbene, morin hydrate, and quercetin on NF-κB activation in LPS-stimulated HEK293T cells (transfectants)

Human embryonic kidney cells (HEK293T) were cultured in DMEM medium supplemented with10% fetal bovine serum. The cells were seeded into 12-well Costar plates (Corning) at a density of 2x10^5^ cells/well and incubated overnight (12 h) in an incubator in a humidified, 5% CO_2_/95% air atmosphere at 37°C. After 12 h, the cells (HEK293T) were transfected with Flag-TLR4 wild type along with pELAM-luciferase (NF-κB reporter; 500 ng/well) and β-gal reporter plasmids (100 ng/well) plus MD-2 and CD14 constructs (3 ng/well of each) for 20 h as described previously [19]. After 20 h, the cells were washed with 1 x PBS, and pretreated with medium only or 40 μM of resveratrol, pterostilbene, morin hydrate or quercetin for 1 h, and washed again with 1 x PBS. The washed cells were stimulated with LPS (5 ng/well) for 5 h, then cells were lysed in 1 x reporter assay lysis buffer (Promega, Madison, WI, USA), and β-galactosidase and luciferase activities (Promega, Madison, WI, USA) were measured by Berthold LB 9507 Luminometer (Berthold Technologies, Oak Ridge, TN, USA). β-galactosidase was used as a control plasmid for normalizing transfection efficiencies and luciferase activities. The relative luminescence activity was calculated by normalizing each sample’s luciferase activity for constitutive β-galactosidase activity measured within the same sample, and represented as “relative luciferase units” (RLU) as described earlier [19]. Based on the expression of β-galactosidase activity in individual cells for each compound, the transfection efficiency was consistently >90%, as assessed by in situ β-galactosidase by using staining kit of Stratagene (Stratagene, La Jolla, CA, USA). Cell viability was >95% in all the treatments [20].

### Effects of resveratrol, pterostilbene, morin hydrate, nicotinic acid, (niacin), and quercetin on expression of TNF-α, IL-1β, IL-6, and iNOS genes in LPS-stimulated RAW 264.7 cells

RAW 264.7 cells (1x10^6^ cells/well in 1.0 mL medium) were allowed to adhere to the bottom of 100 x 20 mm tissue culture plates for 2 h, the supernatants were removed, and cells were washed extensively with medium three times. The cells were treated with resveratrol, pterostilbene, morin hydrate, nicotinic acid, quercetin, δ-tocotrienol, or riboflavin (16 μM of each compound, dissolved in 0.2 % DMSO) for 2 h. Then all the wells were challenged with LPS (10 ng/well; 400 μL), and incubated at 37°C in an incubator in a humidified, 5% CO_2_/95% air atmosphere for 4 h.

Similarly, thioglycolate-elicited peritoneal macrophages were prepared from 8-week-old C57BL/6 and BALB/c female mice as described previously [16,17]. Macrophages from both groups (1x10^7^/well) were allowed to adhere for 2 h to tissue culture wells, cells were treated with resveratrol, pterostilbene, morin hydrate, nicotinic acid, quercetin, δ-tocotrienol, or riboflavin (40 μM of each compound, dissolved in 0.4 % DMSO) for 2 h. After 2 h, wells were challenged with LPS (10 ng/well in 400 μL medium), and incubated at 37°C in a humidified, 5% CO_2_/95% air atmosphere for 4 h. After LPS stimulation, assay mixtures of all treatments were centrifuged at 2,000 rpm for 20 min. The cells were then harvested, and the total cellular RNA was extracted from each pellet with RNeasy mini kit (QIAGEN Sciences, German town, MD, USA) according to the instructions of the manufacturer, and gene analyses were performed on the purified RNA after its conversion to DNA as described earlier [5,21]. The cDNA for each treatment was amplified and analyzed by real-time polymerase chain reaction (RT-PCR) to quantify gene expression of TNF-α, IL-1β, IL-6 and iNOS by using 1-step RT-PCR kit (QIAGEN Science, Chatsworth, CA, USA) according to the manufacturer’s instructions [22]. The viability of LPS-stimulated thioglycolate-elicited peritoneal macrophages and RAW 264.7 cells was determined by trypan blue dye exclusion method or a quantitative colorimetric assay with 3-(4,5)-dimethylthiozol-2,5-tetrazolium bromide (MTT) as described previously [20].

### Statistical analyses

Stat View software (version 4.01, Abacus Concepts, Berkeley, CA) was used for the analyses of treatment-mediated effects as compared to control group. Treatment-mediated differences were detected with a two-way ANOVA, and when the F test indicated a significant effect, differences between the means were analyzed by a Fisher’s protected least significant difference test. Data were reported as means ± SD in text and Tables. The statistical significance level was set at 5% (***P*** < 0.05).

## Results

The results are presented in the same Figure in two different formats: ‘A’ shows the actual measured or calculated values for each treatment and controls, and ‘B’ shows the percent change based on actual calculated values for each treatment compared to control values.

### Inhibitory effects of resveratrol, pterostilbene, morin hydrate, nicotinic acid, (niacin), and quercetin on chymotrypsin-like, trypsin-like, and post-glutamase proteasomal activities in RAW 264.7 whole cells

Twenty S (20 S) proteasomes contain three distinct subunits (X, Y, and Z), with well characterized protease sites; subunits X (β5), Y (β1), and Z (β2) display chymotrypsin-like, trypsin-like, and post-glutamase activities, respectively [22,23]. This series of experiments was designed to determine whether resveratrol, pterostilbene, morin hydrate, and nicotinic acid (niacin) act as proteasome inhibitors, by measuring their effects on chymotrypsin-like, trypsin-like and post-glutamase proteasomal activities. Quercetin, which was previously shown to inhibit proteasomal enzymatic activity, was used as a positive control [5].

**Figure 2:**
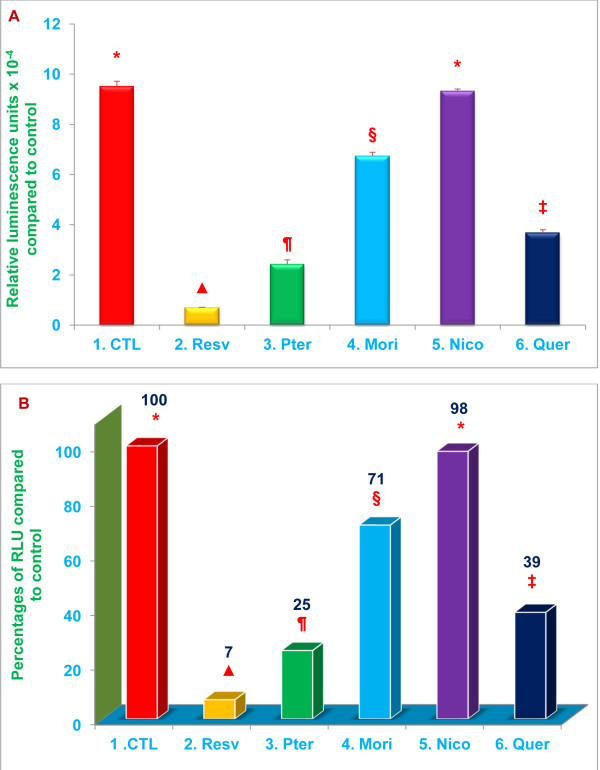
**Resveratrol, pterostilbene, morin hydrate, and quercetin inhibit chymotrypsin-like proteasomal activity.** Relative chymotrypsin-like activity was quantitated by measuring luminescence after treatment of RAW 264.7 whole cells (1x10^4^ cells/well) with 20 μM of resveratrol, pterostilbene, morin hydrate, nicotinic acid, or quercetin. Cell viability exceeded 95% in all the treatments. Data are means ± SD, *n* = 3 per treatment, and triplicate analyses of each sample were performed. Values in a column not sharing a common symbol are significantly different at ***P*** < 0.05. CTL = Control (Medium + RAW 264.7 whole cells + 0.2 % DMSO); Resv = Resveratrol; Pter = Pterostilbene; Mori = Morin hydrate; Nico = Nicotinic acid; Quer = Quercetin-HCL. Figure A = Actual Relative Luminescence Unit (RLU) values. Figure B = Percentages of actual RLU values compared to control.

The chymotrypsin-like activity in RAW 264.7 cells was inhibited by resveratrol, pterostilbene, morin hydrate, and quercetin (positive control), but not by nicotinic acid (Table 1). Inhibition by morin hydrate and quercetin was highly dose-dependent, whereas inhibition by resveratrol and pterostilbene was mildly dose-dependent for the concentrations tested (10 μM to 320 μM; Table 1). Chymotrypsin-like activity was inhibited by >90% and >70% (***P*** < 0.02) by resveratrol and pterostilbene, respectively, at the lowest concentrations tested (10 μM; Table 1). Quercetin and morin hydrate were somewhat less potent, inhibiting chymotrypsin-like activity by 61% and 29%, respectively, (***P*** < 0.05) at 20 μM concentration (Table 1). Thus, of the compounds tested, resveratrol appears to be the most potent inhibitor of chymotrypsin-like activity. For subsequent experiments, we selected a single concentration (20 μM) for testing each of these compounds. The inhibitory effect of these compounds on chymotrypsin-like activity at a concentration of 20 μM is presented in Figure 2.

**Table 1:** **Resveratrol, pterostilbene, morin hydrate, and quercetin inhibit chymotrypsin-like proteasomal activity**^**1**^

NO	Assay mixture	Resveratrol	Pterostilbene	Morin Hydrate	Nicotinic acid	Quercetin*
Average digital values of relative luminescence units (RLU)						
1	Medium + cells = A	87458	87458	87458	87458	87458
2	A + DMSO control^2^	95222 ± 2014 (100)^3^	95222 ± 2014(100)^3^	95750 ± 2014 (100)^3^	95750 ± 2014 (100)^3^	95750 ± 2014 (100)^3^
3	10 μM	7376 ± 107 (8)	27543 ± 947 (29)	89557 ± 1958 (94)	95240 ± 2020 (99)	61823 ± 2206 (65)
4	20 μM	6996 ± 143 (7)	24177 ± 1775 (25)	67338 ± 1626 (71)	95342 ± 1896 (100)	36772 ± 1201 (39)
5	40 μM	2742 ± 138 (3)	23574 ± 1126 (25)	55078 ± 1444 (58)	94232 ± 1962 (98)	19805 ± 732 (21)
6	80 μM	2261 ± 124 (2)	21639 ± 1667 (23)	41178 ± 1099 (43)	94155 ± 1926 (98)	17478 ± 783 (18)
7	160 μM	1688 ± 121 (2)	19483 ± 1207 (20)	33814 ± 134 (36)	95165 ± 1966 (99)	13827 ± 621 (15)
8	320 μM	1590 ± 92 (2)	13380 ± 1341 (14)	27448 ± 88 (29)	94124 ± 2038 (98)	5896 ± 321 (6)

^1^ Chymotrypsin-like activity was quantitated by measuring luminescence after treating RAW 264.7 whole cells (1x10^4^) with various concentrations (10 μM - 320 μM) of resveratrol, pterostilbene, morin hydrate, nicotinic acid, or quercetin as described in Methods. The relative luminescence units (RLU) of assays were read in a Promega Plate Luminometer.

^2^Medium + RAW 264.7 whole cells + 0.2 % dimethyl sulfioxide (DMSO) was used as control. ^3^Percentages based on digital values of RLU compared to control are in parenthesis. *Similar results were reported in Lipids in Health and Disease 2011, **10**:177.

**Figure 3:**
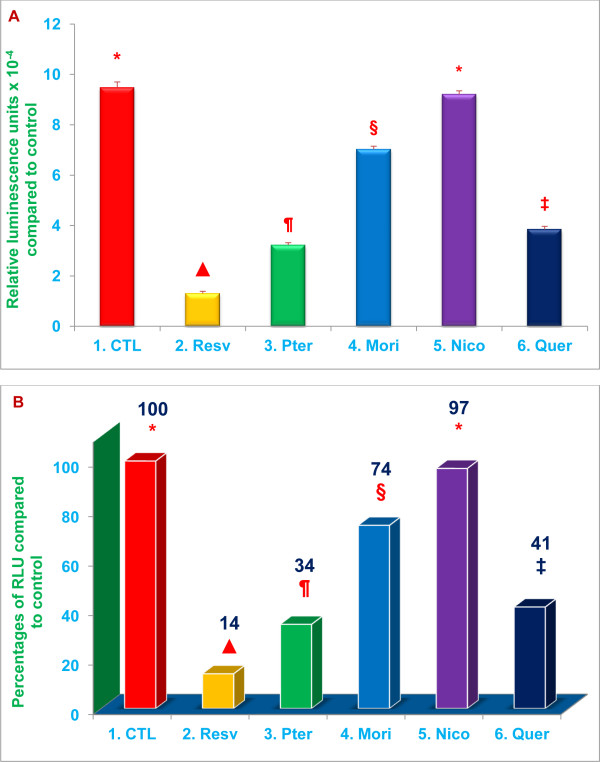
**Resveratrol, pterostilbene, morin hydrate, and quercetin inhibit trypsin-like proteasomal activity.** Relative trypsin-like activity was quantitated by measuring luminescence after treatment of RAW 264.7 whole cells (1x10^4^ cells/well) with 20 μM of resveratrol, pterostilbene, morin hydrate, nicotinic acid, or quercetin. Cell viability exceeded 95% in all the treatments. Data are means ± SD, *n* = 3 per treatment, and triplicate analyses of each sample were performed. Values in a column not sharing a common symbol are significantly different at ***P*** < 0.05. CTL = Control (Medium + RAW 264.7 whole cells + 0.2 % DMSO); Resv = Resveratrol; Pter = Pterostilbene; Mori = Morin hydrate; Nico = Nicotinic acid; Quer = Quercetin-HCL. Figure A = Actual Relative Luminescence Unit (RLU) values. Figure B = Percentages of actual RLU values compared to control.

**Figure 4:**
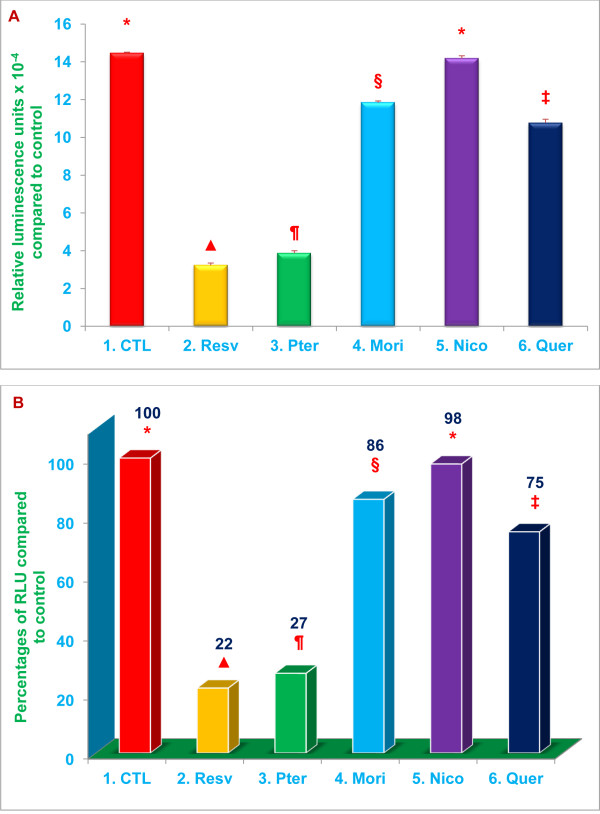
**Resveratrol, pterostilbene, morin hydrate, and quercetin inhibit post-glutamase proteasomal activity.** Relative post-glutamase activity was quantitated by measuring luminescence after treatment of RAW 264.7 whole cells (1x10^4^ cells/well) with 20 μM of resveratrol, pterostilbene, morin hydrate, nicotinic acid, quercetin or medium alone (control). Cell viability exceeded 95 % in all the treatments. Data are means ± SD, n = 3 per-treatment, and triplicate analyses of each sample were performed. Values in a column not sharing a common symbol are significantly different at ***P*** <0.05. CTL = Control (Medium + RAW 264.7 whole cells + 0.2 % DMSO); Resv = resveratrol; Pter = pterostilbene; Mori = morin hydrate; Nico = nicotinic acid; Quer = quercetin-HCL. Figure A = actual relative luminescence unit (RLU) values. Figure B = Percentages of actual RLU values compared to control.

Proteasomal trypsin-like activity was inhibited by 86%, 66 % (***P*** < 0.02), 26 %, and 59 % (***P*** < 0.05) with 20 μM resveratrol, pterostilbene, morin hydrate, and quercetin, respectively (Figure 3). Similarly, post-glutamase activity was inhibited by 78 %, 73 % (***P*** < 0.02), 14 % and 25 % (***P*** < 0.05) with 20 μM resveratrol, pterostilbene, morin hydrate, and quercetin, respectively (Figure 4). Nicotinic acid failed to inhibit both trypsin-like and post-glutamase activities of the proteasome (Figures 3 and 4). The data presented in Figures 2, 3 and 4 demonstrate that, of the compounds tested, resveratrol and pterostilbene are the most potent inhibitors of all three proteasomal enzymatic activities.

### Resveratrol, pterostilbene, morin hydrate, nicotinic acid, and quercetin inhibit production of NO by RAW 264.7 cells stimulated with LPS alone or LPS plus interferon-γ (IFN-γ)

**Figure 5:**
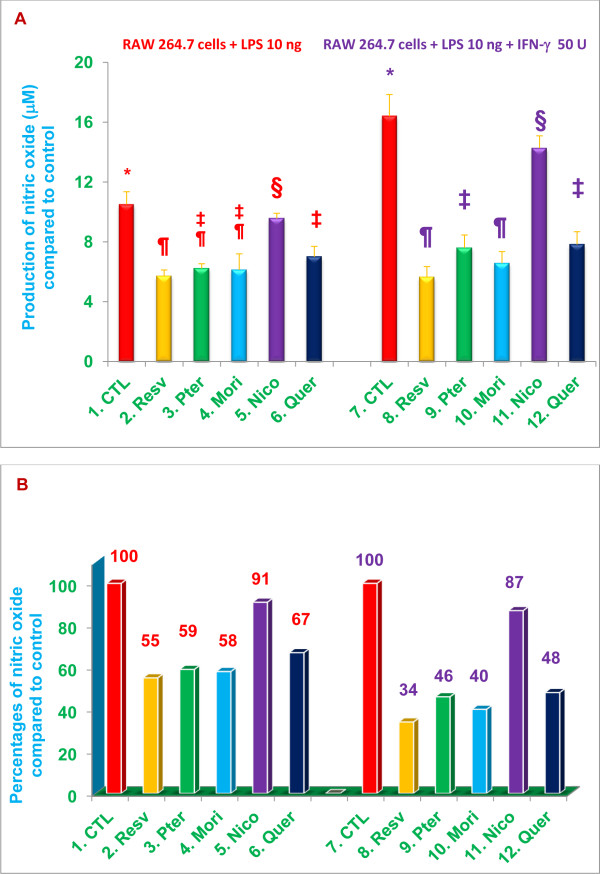
**Resveratrol, pterostilbene, morin hydrate, and quercetin inhibit production of NO by RAW 264.7 cells treated with LPS alone, or LPS plus interferon-γ (IFN-γ).** NO levels were measured in supernatants of RAW 264.7 treated with resveratrol, pterostilbene, morin hydrate, nicotinic acid, quercetin (16 μM) or medium alone (control) for 1 h, then challenged with LPS (10 ng/well) alone, LPS + IFN-γ (10 ng + 50 U/well) or medium alone (negative control) for 36 h. Cell viability exceeded 95% for all treatments. Data are means ± SD, *n* = 3 per treatment, and triplicate analyses of each sample were performed. Values in a column not sharing a common symbol are significantly different at ***P*** < 0.05. CTL = Control (Medium + RAW 264.7 cells + 0.2% DMSO); Resv = Resveratrol; Pter = Pterostilbene; Mori = Morin hydrate; Nico = Nicotinic acid; Quer = Quercetin-HCL. Figure A = Actual Relative Luminescence Unit (RLU) values. Figure B = Percentages of actual RLU values compared to control.

We recently reported that δ-tocotrienol, quercetin, riboflavin, and dexamethasone inhibited NO production by LPS-stimulated RAW 264.7 cells and thioglycolate-elicited peritoneal macrophages derived from C57BL/6 and BALB/c mice [5]. Consequently, we were interested in determining whether resveratrol, pterostilbene, morin hydrate, and nicotinic acid would have similarly suppressive effects on NO responses to LPS-stimulation. RAW 264.7 cells were pretreated with resveratrol, pterostilbene, morin hydrate, niacin, or quercetin (positive control; 16 μM of each compound) for 60 min, followed by stimulation with LPS alone (10 ng/well) or LPS plus IFN-γ (10 ng + 50 U/well) at room temperature for 36 h; NO was measured in cell culture supernatants. IFN-γ was used because it augments the NO response of LPS-stimulated macrophages [21]. Significant (***P***<0.05) inhibition of NO production by RAW 264.7 cells stimulated with LPS alone or LPS plus IFN-γ was attained with resveratrol (45%), pterostilbene (41%), morin hydrate (42%), and quercetin (33%), but not with nicotinic acid (Figure 5A,B). The extent of NO suppression did not differ markedly for the four compounds, but inhibition of NO production was more pronounced in RAW 264.7 cells stimulated with LPS plus IFN-γ (66%, 54%, 60%, 52% respectively; *P*<0.02) compared to those stimulated with LPS alone (Figure 5A,B).

### Resveratrol, pterostilbene, morin hydrate, nicotinic acid, and quercetin inhibit secretion of TNF-α by LPS-stimulated RAW 264.7 cells

It is well established that TNF-α is among the earliest and most important pro-inflammatory cytokines produced in response to a variety of inflammatory stimuli, and we have previously demonstrated that a number of naturally-occurring proteasome inhibitors suppress secretion of TNF-α by LPS-stimulated macrophages *in vitro* and *in vivo*[5,22,23]. Consequently, this series of experiments was conducted to determine whether resveratrol, pterostilbene, morin hydrate, nicotinic acid and quercetin had similarly suppressive effects on TNF-α secretion by LPS-stimulated RAW 264.7 cells.

**Figure 6:**
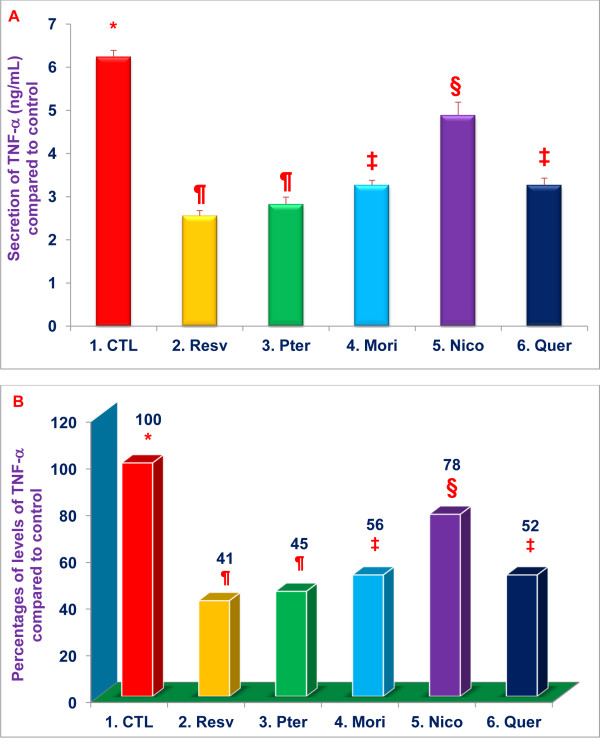
**Resveratrol, pterostilbene, morin hydrate, nicotinic acid and quercetin inhibit TNF-α secretion by LPS treated RAW 264.7 cells.** TNF-α levels were measured in supernatants of RAW 264.7 treated with resveratrol, pterostilbene, morin hydrate, nicotinic acid, quercetin (16 μM) or medium alone (control) for 1 h, then challenged with LPS (10 ng/well; 400 μL) or medium alone for 4 h. Cell viability exceeded 95% for all treatments. Data are means ± SD, *n* = 3 per treatment, and triplicate analyses of each sample were performed. Values in a column not sharing a common symbol are significantly different at ***P*** < 0.05. CTL = Control (Medium + RAW 264.7 cells + 0.2 % DMSO); Resv = Resveratrol; Pter = Pterostilbene; Mori = Morin hydrate; Nico = Nicotinic acid; Quer = Quercetin-HCL. Figure A = Actual Relative Luminescence Unit (RLU) values. Figure B = Percentages of actual RLU values compared to control.

RAW 264.7 macrophage-like cells were pretreated for 1 h with resveratrol, pterostilbene, morin hydrate, nicotinic acid, or quercetin (16 μM of each), followed by stimulation with LPS (10 ng/well) and measurement of TNF-α in supernatants 4 h after LPS stimulation. Resveratrol (59 %), pterostilbene (55 %), morin hydrate (44 %), and quercetin, (48 %) significantly (***P*** < 0.02) inhibited LPS-induced secretion of TNF-α, whereas, nicotinic acid (22 %) produced only (***P*** < 0.05) moderate inhibition (Figure 6). We previously demonstrated that both chymotrypsin-like and trypsin-like enzymatic proteasomal activity of the proteasome had to be inhibited in order to suppress TNF-α secretion by LPS stimulated macrophages [22,23]. Thus, our findings that these compounds inhibited both chymotrypsin-like and trypsin-like activities of the proteasome (Figures 2 and 3) are consistent with their ability to inhibit TNF-α secretion by LPS-stimulated RAW 264.7 cells (Figure 6).

### Inhibition of NF-κB activation in LPS-stimulated HEK293T cells by resveratrol, pterostilbene, morin hydrate, and quercetin

We recently hypothesized that increased NO levels, associated with ageing, may be attributable to increased degradation of phosphorylated IκB by ageing proteasomes, resulting in enhanced NF-κB activation. In studying the potential mechanisms by which a variety of proteasome inhibitors suppress overproduction of inflammatory cytokines associated with ageing, we determined that they inhibited NF-κB activation [5]. NF-κB is normally complexed with IκB in the cytoplasm and becomes activated when IκB is phosphorylated, ubiquitinated, and subsequently degraded by the proteasome [24]. Once it dissociates from IκB, NF-κB translocates to the nucleus where it binds to promoter sites of pro-inflammatory genes such as TNF-α and iNOS [24].

**Figure 7:**
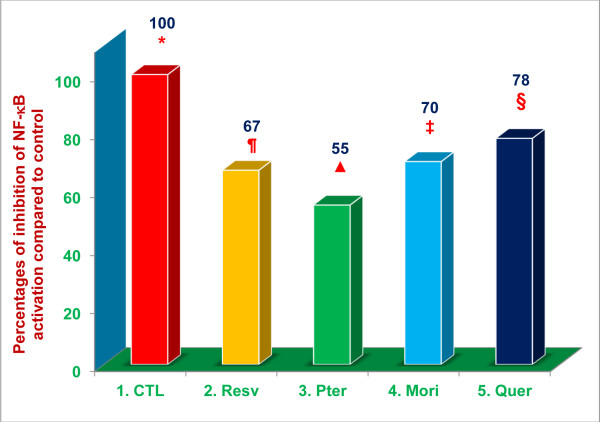
**Resveratrol, pterostilbene, morin hydrate, and quercetin inhibit NF-κB activation in LPS-stimulated HEK293T cells.** Relative luminescence of an NF-κB reporter compared to a β-galactosidase reporter (control) was measured to determine the extent of NF-κB activation in HEK293T cells treated with resveratrol, pterostilbene, morin hydrate, quercetin (16 μM) or medium alone for 1 h, then challenged with LPS (5 ng/well) for 5 h. Cell viability was >95% in all the treatments. Data are means ± SD, *n* = 2 per treatment, and duplicate analyses of each sample were performed. Values in a column not sharing a common symbol are significantly different at ***P*** < 0.05. CTL = Control (Medium + HEK293T cells + 0.4% DMSO); Resv = Resveratrol; Pter = Pterostilbene; Mori = Morin hydrate; Quer = Quercetin-HCL. Data presented represent the percent inhibition of NF-κB activation compared to control.

All of the proteasome inhibitors that we have tested to date acted by blocking the degradation of phosphorylated and ubiqutinated IκB by the proteasome, thereby preventing NF-κB activation [5-7,25]. For this series of experiments we wanted to determine if resveratrol, pterostilbene, and morin hydrate, behave similarly to previously tested proteasome inhibitors, by inhibiting NF-κB activation in LPS-stimulated HEK293T cells; quercetin, was used as a positive control. Significant inhibition (***P*** < 0.02) of LPS-induced NF-κB activation, compared to medium controls, was achieved with resveratrol (33 %), pterostilbene (45 %), morin hydrate (30 %), and quercetin (22 %) compared to controls (Figure 7). Thus, the capacity of each of these compounds to inhibit production of NO, and secretion of TNF-α in response to LPS-stimulation, appears to be dependent upon their ability to suppress LPS-induced NF-κB activation.

### Inhibitory effects of resveratrol, pterostilbene, morin hydrate, nicotinic acid, and quercetin on expression of TNF-α, IL-1β, IL-6, and iNOS genes in LPS-stimulated RAW 264.7 cells

In an earlier study, we demonstrated that several naturally-occurring proteasome inhibitors (quercetin, riboflavin, and δ-tocotrienol) suppress expression of TNF-α and iNOS genes in LPS-stimulated thioglycolate-elicited peritoneal macrophages derived from several strains of mice [5]. Other inflammatory cytokines (e.g. IL-1β. IL-6), have also been implicated in age-related diseases, so the following experiment was conducted to determine whether resveratrol, pterostilbene, morin hydrate, and nicotinic acid have the capacity to suppress expression of IL-6, in addition to TNF-α and iNOS genes, in LPS-stimulated RAW 264.7 cells.

RAW 264.7 cells (1x10^7^ cells/well) were allowed to adhere for 4 h in the wells, and after 4 h, cells were treated with resveratrol, pterostilbene, morin hydrate, or nicotinic acid (16 μM of each compound in 0.2% DMSO) for 1 h; quercetin (16 μM) was used as a positive control. All wells were subsequently challenged with LPS (10 ng/well), and incubated at 37 °C in an incubator for 4 h. Total RNA was extracted using the RNeasy mini kit, and analyzed by RT-PCR to quantify gene expression of various cytokines. TNF-α, IL-1β, IL-6, and iNOS gene expression in LPS-stimulated RAW 264.7 cells was significantly (***P***<0.05) inhibited by all compounds tested, with one exception; nicotinic acid failed to inhibit expression of the TNF-α gene (Table 2).

**Table 2:** **Resveratrol, pterostilbene, morin hydrate, nicotinic acid, and quercetin inhibit expression of TNF-α, IL-1β, IL-6, and iNOS genes in LPS-stimulated RAW 264.7 cells**^**1**^

NO	Treatments	RT-PCR data (*Ratios of optical density of gene expression of cytokines/β-actin).			
		TNF-α	IL-1β	IL-6	iNOS
1	Media + Cells = A	0.05	0.05	0.1	0.2
2	A + LPS (10 ng/well) = B	0.83	1.35	0.93	1.12
3	B + 0.2 % DMSO = C	0.80 ± 0.03^a^ (100)^2^	1.32 ± 0.03^a^ (100)^2^	0.96 ± 0.03^a^ (100)^2^	1.04 ± 0.02^a^ (100)^2^
4	C + Resveratrol (16.0 μM)	0.18 ± 0.02^e^ (23)	0.51 ± 0.03^e^ (39)	0.21 ± 0.0^e^ (22)	0.65 ± 0.02^c^ (63)
5	C + Pterostilbene (16.0 μM)	0.38 ± 0.03^d^ (48)	0.75 ± 0.03^d^ (57)	0.62 ± 0.03^c^ (65)	0.92 ± 0.03^b^ (88)
6	C + Morin hydrate (16.0 μM)	0.55 ± 0.03b (69)	0.92 ± 0.04c (70)	0.63 ± 0.02c (66)	0.53 ± 0.03d (51)
7	C + Nicotinic acid (16.0 μM)	0.76 ± 0.05^a^ (95)	1.12 ± 0.02^b^ (85)	0.75 ± 0.03^b^ (78)	0.66 ± 0.05^c^ (64)
8	C + Quercetin (16.0 μM)	0.45 ± 0.02^c^ (56)	0.22 ± 0.02^f^ (17)	0.25 ± 0.0^d^ (26)	0.37 ± 0.03^e^ (36)

^1^RAW 264.7 cells were treated with resveratrol, pterostilbene, morin hydrate, nicotinic acid, or quercetin (16 μM) for 1 h, followed by treatment with LPS (10 ng/well) for 4 h. Total RNA was extracted, reverse transcribed, and the resulting DNA was amplified and analyzed by real time PCR to quantitate expression of TNF-α, IL-1β, IL-6, and iNOS genes [5,21].

Cell viability was >95% in all treatments. Data are means ± SD, *n* = 3 (triplicate analysis of each sample).

^2^Percentages of *digital values of relative optical density of each cytokine/β-actin of each treatment are in parenthesis. ^a-f^Values in a column not sharing a common superscript letter are significantly different at ***P*** < 0.05.

Quercetin (positive control) inhibited expression of TNF-α, IL-1β, and IL-6 genes by 44%, 83%, and 74%, respectively, compared to controls (Table 2). Of the experimental compounds tested, the most pronounced inhibition of TNF-α, IL-1β, and IL-6 gene expression (77%, 61%, and 78% suppression, respectively; ***P***<0.02) compared to respective controls, was obtained with resveratrol. Inhibition of TNF-α, IL-1β, and IL-6 gene expression was somewhat less pronounced with pterostilbene (52%, 43%, and 35%, respectively; ***P*** < 0.05), and morin hydrate (31%, 30%, and 34% inhibition, respectively) compared to respective controls. In contrast, morin hydrate was the most potent inhibitor of iNOS gene expression (49%; ***P*** <0.05; Table 2). The results of gene expression inhibition studies for TNF-α and iNOS genes were generally consistent with those of secretion of TNF-α and NO production. Thus, these experiments support the conclusion that resveratrol, pterostilbene, and morin hydrate exert anti-inflammatory effects, at least in part, due to their ability to suppress gene expression of TNF-α, iNOS, IL-1β, and IL-6 in LPS-stimulated RAW 264.7 cells. Nicotinic acid, in contrast, did not suppress expression of the TNF-α gene, and its capacity to suppress expression of the IL-1β and IL-6 genes was also relatively modest; it did, however, demonstrate relatively potent inhibition of iNOS gene expression (36%) compared to controls (Table 2).

### Inhibitory effects of resveratrol, pterostilbene, morin hydrate, nicotinic acid, and quercetin on expression of TNF-α, IL-1β, IL-6, and iNOS genes in LPS-stimulated, peritoneal macrophages from 8-week-old C57BL/6 and BALB/c mice

In order to confirm the results obtained with cultured RAW 264.7 cells (Table 2), we decided to determine the effects of resveratrol, pterostilbene, morin hydrate, and nicotinic acid on TNF-α, IL-1β, IL-6 and iNOS gene expression in LPS-stimulated, thioglycolate-elicited peritoneal macrophages from C57BL/6 and BALB/c mice; quercetin was used as a positive control. Cells (1x10^6^/well) were adhered to tissue culture plate for 4 h, after which cells were treated with resveratrol, pterostilbene, morin hydrate, nicotinic acid, or quercetin (40 μM/well in 0.2 % DMSO) for 1 h. LPS (10 ng/well) was then added to each well and incubated for 4 h. After 4 h total cellular RNA was extracted and reverse-transcribed, and gene expression was quantified by RT-PCR [5,21].

Expression of TNF-α, IL-1β, IL-6, and iNOS genes in LPS-stimulated, thioglycolate-elicited peritoneal macrophages from C57BL/6 mice was significantly (***P*** < 0.02) inhibited by resveratrol, pterostilbene, morin hydrate, and quercetin (positive control); nicotinic acid did not significantly suppress expression of any of these genes (Table 3). The most pronounced inhibition of TNF-α, IL-1β, and IL-6 gene expression was attained with resveratrol (72%, 80%, and 82%, respectively) and pterostilbene (69%, 75%, and 81% respectively). Resveratrol (31%), pterostilbene (34 %), morin hydrate (39 %), and quercetin (36 %) were also potent inhibitors of iNOS gene expression (Table 3) compared to respective controls. Nicotinic acid (15%) was only a modest inhibitor of iNOS gene expression (Table 3). Results with peritoneal macrophages from BALB/c mice (Table 4) were almost identical to those attained with C57BL/6 mice (Table 3).

**Table 3:** **Resveratrol, pterostilbene, morin hydrate, nicotinic acid, and quercetin inhibit expression of TNF-α, IL-1β, IL-6, and iNOS genes in peritoneal macrophages from C57BL/6 mice**^**1**^

NO	Treatments	RT-PCR data (Ratios of digital values of optical density of gene expression of cytokines/β-actin).			
		TNF-α	IL-1β	IL-6	iNOS
1	Media + Cells = A	0.05	0.05	0.1	0.2
2	A + LPS (10 ng/well) = B	0.45	5.32	8.67	4.67
3	B + 0.4 % DMSO = C	0.36 ± 0.02^a^ (100)^2^	6.89 ± 2.01^a^ (100)^2^	12.34 ± 1.96^a^ (100)^2^	5.67 ± 1.90^a^ (100)^2^
4	C + Resveratrol (40.0 μM)	0.10 ± 0.01^c^ (28)	1.39 ± 0.04^c^ (20)	2.16 ± 0.60^c^ (18)	3.93 ± 1.50^b^ (69)
5	C + Pterostilbene (40.0 μM)	0.11 ± 0.01^c^ (31)	1.74 ± 0.48^b^ (25)	2.33 ± 0.82^c^ (19)	3.76 ± 1.40^b,c^ (66)
6	C + Morin hydrate (40.0 μM)	0.14 ± 0.05^b^ (39)	1.87 ± 0.49^b^ (27)	3.03 ± 1.03^b^ (25)	3.47 ± 0.03^b,c^ (61)
7	C + Nicotinic acid (40.0 μM)	0.33 ± 0.03^a^ (92)	6.27 ± 0.42^a^ (91)	10.86 ± 0.91^a^ (88)	4.81 ± 0.66^a^ (85)
8	C + Quercetin (40.0 μM)	0.11 ± 0.04^c^ (31)	2.14 ± 0.46^b^ (31)	3.24 ± 0.93^b^ (26)	3.65 ± 1.18^b,c^ (64)

^1^Thioglycolate-elicited peritoneal macrophages from 8-week-old C57BL/6 mice were treated with resveratrol, pterostilbene, morin hydrate, Cell viability was >95% in all the treatments. Data are means ± SD, *n* = 3 (triplicate analysis of each sample).

Cell viability was >95 % in all the treatments. Data are means ± SD, n = 3 (triplicate analysis of each sample).

^2^Percentages of digital values of relative optical density of each cytokine/β-actin of each treatment are in parenthesis.

^a-c^Values in a column not sharing a common superscript letter are significantly different at ***P*** < 0.05.

**Table 4:** **Resveratrol, pterostilbene, morin hydrate, nicotinic acid, and quercetin inhibit expression of TNF-α, IL-1β, IL-6, and iNOS genes in peritoneal macrophages from BALB/c mice**^**1**^

NO	Treatments	RT-PCR data (Ratios of digital values of optical density of gene expression of cytokines/β-actin).			
		TNF-α	IL-1β	IL-6	iNOS
1	Media + Cells = A	0.05	0.05	0.1	0.2
2	A + LPS (10 ng/well) = B	0.62	8.27	14.45	7.76
3	B + 0.4 % DMSO = C	0.74 ± 0.11^a^ (100)^2^	10.68 ± 1.18^a^ (100)^2^	15.68 ± 1.13^a^ (100)^2^	9.85 ± 1.04^a^ (100)^2^
4	C + Resveratrol (40.0 μM)	0.28 ± 0.06^c^ (38)	3.25 ± 0.97^b^ (30)	4.43 ± 1.23^b,c^ (28)	7.18 ± 0.88^b^ (73)
5	C + Pterostilbene (40.0 μM)	0.35 ± 0.08^b^ (47)	3.55 ± 1.00^b^ (33)	5.08 ± 0.76^b,c^ (32)	6.68 ± 1.10^b^ (68)
6	C + Morin hydrate (40.0 μM)	0.32 ± 0.09^c^ (43)	3.75 ± 1.04^b^ (35)	5.68 ± 1.09^b^ (36)	7.28 ± 0.97^b^ (74)
7	C + Nicotinic acid (40.0 μM)	0.65 ± 0.11^a^ (88)	9.74 ± 1.33^a^ (91)	12.76 ± 0.91^a^ (81)	8.17 ± 1.05^a^ (83)
8	C + Quercetin (40.0 μM)	0.30 ± 0.12^c^ (41)	3.63 ± 1.40^b^ (34)	6.10 ± 0.77^b^ (39)	7.12 ± 1.07^b^ (72)

^1^Thioglycolate-elicited peritoneal macrophages from 8-week-old BALB/c mice were treated with resveratrol, pterostilbene, morin hydrate, nicotinic acid, or quercetin (40 μM) for 1 h, followed by treatment with LPS (10 ng/well) for 4 h. Total RNA was extracted, reverse transcribed, and the resulting DNA was amplified and analyzed by real time PCR to quantitate expression of TNF-α, IL-1β, IL-6, and iNOS genes [5,21]. Cell viability was >95 % in all the treatments. Data are means ± SD, n = 3 (triplicate analysis of each sample).

^2^Percentages of digital values of relative optical density of each cytokine/β-actin of each treatment are in parenthesis.

^a-c^Values in a column not sharing a common superscript letter are significantly different at ***P*** < 0.05.

In summary, resveratrol, pterostilbene, and morin hydrate significantly (P <0.02) reduced TNF-α, IL-1β, IL-6 and iNOS mRNA expression in LPS-stimulated RAW 264.7 cells and thioglycolate-elicited peritoneal macrophages derived from C57BL/6 or BALB/c mice. Of the compounds tested, resveratrol and pterostilbene appear to produce slightly more potent inhibition of TNF-α, IL-1β, and IL-6 gene expression than morin hydrate. iNOS gene expression, in contrast, is at least as susceptible to inhibition by morin hydrate, as it is to inhibition by resveratrol and pterostilbene. Nicotinic acid failed to inhibit TNF-α, IL-1β, IL-6 and iNOS gene expression in LPS-stimulated peritoneal macrophages from either C57BL/6 or BALB/c mice, and produced only mild inhibitory effects in LPS-stimulated RAW 264.7 cells.

### Inhibitory effects of δ-tocotrienol and riboflavin on expression of TNF-α, IL-1β, IL-6, and iNOS genes in RAW 264.7 cells and peritoneal macrophages from 8-week-old C57BL/6 and BALB/c mice stimulated with LPS

We have now demonstrated that resveratrol, pterostilbene, morin hydrate, and quercetin have the capacity to suppress gene expression of IL-1β and IL-6, in addition to TNF-α and iNOS genes, in LPS-stimulated RAW 264.7 cells and peritoneal thioglycolate-elicited macrophages derived from C57BL/6 and BALB/c mice (Tables 2,3,4). We had previously studied the ability of other proteasome inhibitors (e.g. δ-tocotrienol and riboflavin) to inhibit expression of TNF-α and iNOS genes under a variety of conditions [5], but had not tested their ability to inhibit IL-1β, and IL-6 gene expression. Consequently, this series of experiments was designed to determine whether δ-tocotrienol and riboflavin would produce results comparable to resveratrol, pterostilbene, morin hydrate, and inhibit IL-1β, and IL-6 gene expression in LPS-stimulated macrophages using identical conditions to those described above for Tables 2,3 and 4.

Expression of TNF-α, IL-1β, IL-6, and iNOS genes in LPS-stimulated RAW 264.7 cells and peritoneal macrophages from both C57BL/6 and BALB/c mice was significantly (***P***<0.05) inhibited by both δ-tocotrienol and riboflavin (Table 5). For virtually all genes and all cells tested, the extent of inhibition attained with δ-tocotrienol was generally greater than that attained with riboflavin. Inhibition of iNOS in BALB/c mice was the only exception; the degree of inhibition by δ-tocotrienol and riboflavin was comparable. Similarly, the extent to which δ-tocotrienol and riboflavin inhibited IL-1β and IL-6 gene expression was comparable, or slightly greater than that observed for TNF-α gene expression in all cell systems tested. Thus, the anti-inflammatory properties of δ-tocotrienol and riboflavin are not limited to their ability to suppress TNF-α and iNOS gene expression; they also suppress expression of IL-1β, and IL-6 genes in LPS-stimulated RAW 264.7 cells and macrophages derived from C57BL/6 and BALB/c mice

**Table 5:** **δ-Tocotrienol and riboflavin inhibit expression of TNF-α, IL-1β, IL-6, and iNOS genes in LPS-stimulate RAW 264.7 cells, and peritoneal macrophages derived from C57BL/6 or BALB/c mice**^**1**^

NO	Treatments	RT-PCR data (Ratios of digital values of optical density of gene expression of cytokines/β-actin).			
		**TNF-α**	**IL-1β**	**IL-6**	**iNOS**
**RAW 264.7 cells.**					
1	Media + Cells = A	0.05	0.05	0.1	0.2
2	A + LPS (10 ng/well) = B	0.83	1.35	0.98	1.12
3	B + 0.2 % DMSO = C	0.80 ± 0.03^a^ (100)^2^	1.32 ± 0.03^a^ (100)^2^	0.96 ± 0.03^a^ (100)^2^	1.04 ± 0.02^a^ (100)^2^
4	C + δ-Tocotrienol (16.0 μM)	0.32 ± 0.03^c^ (40)	0.33 ± 0.03^c^ (25)	0.41 ± 0.04^c^ (43)	0.42 ± 0.04^c^ (40)
5	C + Riboflavin (16.0 μM)	0.64 ± 0.04^b^ (80)	0.95 ± 0.03^b^ (72)	0.82 ± 0.04^b^ (85)	0.76 ± 0.03^b^ (73)
**8-week-old C57BL/6**					
1	Media + Cells (macrophages) = A	0	0	0	0
2	A + LPS (10 ng/well) = B	0.35	5.32	11.67	4.97
3	B + 0.4 % DMSO = C	0.36 ± 0.02^a^ (100)^2^	6.89 ± 0.86^a^ (100)^2^	12.34 ± 1.1^a^ (100)^2^	5.67 ± 0.67^a^ (100)^2^
4	C + δ-Tocotrienol (40.0 μM)	0.16 ± 0.08^c^ (45)	2.34 ± 0.42^c^ (34)	1.98 ± 0.24^c^ (16)	2.12 ± 0.56^c^ (56)
5	C + Riboflavin (40.0 μM)	0.22 ± 0.02^b^ (61)	4.15 ± 0.68^b^ (60)	7.23 ± 0.98^b^ (59)	4.16 ± 0.53^b^ (73)
**8-week-old BALB/c**					
1	Media + Cells (macrophages) = A	0	0	0	0
2	A + LPS (10 ng/well) = B	0.62	8.27	14.45	7.76
3	B + 0.4 % DMSO = C	0.74 ± 0.05^a^ (100)^2^	10.67 ± 1.24^a^ (100)^2^	15.67 ± 1.45^a^ (100)^2^	9.85 ± 0.88^a^ (100)^2^
4	C + δ-Tocotrienol (40.0 μM)	0.39 ± 0.01^c^ (53)	4.16 ± 0.36^c^ (39)	3.45 ± 0.28^c^ (22)	6.64 ± 0.86^b^ (67)
5	C + Riboflavin (40.0 μM)	0.54 ± 0.03^b^ (73)	6.45 ± 0.91^b^ (60)	4.73 ± 0.22^b^ (30)	7.64 ± 0.67^b^ (78)

^1^RAW 264.7 cells or thioglycolate-elicited peritoneal macrophages from 8-week-old C57BL/6 or BALB/c mice were treated with δ-tocotrienol or riboflavin (16 μM or 40 μM) for 1 h, followed by treatment with LPS (10 ng/well) for 4 h. Total RNA was extracted, reverse transcribed, and the resulting DNA was amplified and analyzed by real time PCR to quantitate expression of TNF-α, IL-1β, IL-6, and iNOS genes [5,21].

Cell viability was >95 % in all the treatments. Data are means ± SD, n = 3 (triplicate analysis of each sample).

^2^Percentages of digital values of relative optical density of each cytokine/β-actin of each treatment are in parenthesis. ^a-c^Values in a column not sharing a common superscript letter are significantly different at P < 0.05.

### Inhibitory effects of δ-tocotrienol combined with resveratrol, pterostilbene, morin hydrate, nicotinic acid, or quercetin on TNF-α secretion and nitric oxide (NO) production by LPSstimulated peritoneal macrophages from C57BL/6 mice

The capacity of δ-tocotrienol, quercetin, riboflavin, resveratrol, pterostilbene, morin hydrate and nicotinic acid to inhibit TNF-α secretion and NO production by LPS-stimulated RAW 264.7 cells and macrophages derived from C57BL/6 and BALB/c has been established. In a previous publication we also demonstrated that diet supplementation with a combination of δ-tocotrienol plus either quercetin or riboflavin produced further decreases in inflammation compared to treatment with any of these agents alone in chickens [6]. Thus, we wanted to determine whether combined treatment of LPS-stimulated peritoneal macrophages with δ-tocotrienol plus resveratrol, pterostilbene, morin hydrate, quercetin, riboflavin or nicotinic acid would produce further reductions in TNF-α secretion and NO production compared to treatment with each of these agents alone. The assay conditions were identical to those described earlier [5]. As shown in Table 6, when each of the compounds was tested individually, there was significant inhibition of TNF-α secretion and NO production by LPS stimulated macrophages compared to controls (rows 4–10 vs. row 3), as anticipated. Importantly, however, the extent to which TNF-α secretion and NO production were inhibited by resveratrol, pterostilbene, morin hydrate, quercetin, riboflavin and nicotinic acid was increased when they were combined with δ-tocotrienol (Table 6; compare row 12 vs. 5; 13 vs. 6; 14 vs. 7, etc). These findings suggest additive or synergistic effects between these compounds, and confirm our earlier findings that combining δ-tocotrienol with either quercetin or riboflavin reduced markers of inflammation in chickens [6].

**Table 6:** **Inhibitory effects of quercetin, riboflavin, resveratrol, pterostilbene, morin hydrate and nicotinic acid with or without δ-tocotrienol on the secretion of TNF-α and production of nitric oxide by LPS-induced peritoneal macrophages from C57BL/6 mice**^**1**^

NO	Assay mixture	Concentration	Serum concentration	Serum concentration
		in μM	of TNF-α (pg/mL)	of nitric oxide (μM)
1	Medium + Cells = A		0	0
2	A + LPS (10 ng/well) = B		1098.87	28.89
3	B + 0.2 % DMSO^2^ = C		1056.23 (100)^3^	28.54 (100)^3^
4	C + δ-Tocotrienol = D	5.0	674.56 (64)	18.98 (67)
5	C + Quercetin	40.0	589.98 (56)	20.43 (72)
6	C + Riboflavin	40.0	502.87 (58)	22.54 (79)
7	C + Resveratrol	40.0	542.76 (51)	17.56 (61)
8	C + Pterostilbene	40.0	567.34 (54)	16.87 (59)
9	C + Morin hydrate	40.0	597.42 (57)	19.65 (69)
10	C + Nicotinic acid	40.0	727.71 (69)	23.89 (84)
11	B + 0.2 % DMSO = C		1062.45 (100)	27.98 (100)
12	D + Quercetin	40.0	432.87 (41)	14.76 (53)
13	D + Riboflavin	40.0	465.21 (44)	17.43 (62)
14	D + Resveratrol	40.0	346.76 (33)	12.67 (45)
15	D + Pterostilbene	40.0	412.45 (39)	13.25 (47)
16	D + Morin hydrate	40.0	432.67 (41)	15.32 (55)
17	D + Nicotinic acid	40.0	588.56 (55)	18.96 (68)

^1^Thioglycolate-elicited peritoneal macrophages from 8-week-old C57BL/6 mice were pre treated with quercetin, riboflavin, resveratrol, pterostilbene, morin hydrate, or nicotinic acid (40 μM) with or without δ-tocotrienol (5 μM) for 1 h, followed by treatment with LPS (10 ng/well). After 4 h (TNF-α) or 36 h (NO) of LPS challenge, supernatants were collected and assayed for TNF-α and NO, respectively. Cell viability was >95% in all the treatments.

^2^DMSO = dimethyl sulfuoxide.

^3^Percentages based on actual values of TNF-α (pg/mL) and NO (μM), compared to controls, are in parentheses.

In summary, the collective results presented in this manuscript strongly support the concept that the inflammatory response of macrophages to LPS, one of the most potent inflammatory stimuli identified to date, can be markedly suppressed by a variety of proteasome inhibitors that have been FDA approved for human consumption.

## Discussion

The key findings of the current study are that resveratrol, pterostilbene and morin hydrate inhibit proteasomal enzymatic activity, as well as the capacity of macrophages to produce inflammatory cytokines and NO in response to LPS stimulation. First, we demonstrated that all three of these compounds inhibited chymotrypsin-like, trypsin-like, and post-glutamase proteasome enzymatic activity of cultured RAW 264.7 macrophage like cells. Next we demonstrated that these three compounds inhibited TNF-α secretion and nitric oxide (NO) production by LPS-stimulated RAW 264.7 cells, and also inhibited expression of TNF-α, IL-1β, IL-6, and iNOS genes in LPS-stimulated RAW 264.7 cells and LPS-stimulated thioglycolate-elicited peritoneal macrophages derived from both C57BL/6 and BALB/c mice. Finally, we provided a mechanistic explanation for these findings, as all three compounds inhibited NF-κB activation in LPS-stimulated HEK293T cells. NF-κB enhances transcription of TNF-α, IL-1β, IL-6, and iNOS genes by binding to promoter sites, so the capacity of resveratrol, pterostilbene and morin hydrate to inhibit NF-κB activation would be expected to suppress transcription of these genes in response to LPS stimulation [26].

As described previously, polyphenols and δ-tocotrienol act as proteasome inhibitors and thus inhibit inflammation [5]. Proteasome inhibitors can exert their effects by impacting transcription factors such as NF-κB, thereby altering gene expression levels, and by altering the degradation of ubiquitinated proteins targeted for processing by the proteasome [24]. Therefore, compounds that target the proteasome affect inflammatory processes and several other biological functions. To the best of our knowledge, this is the first report demonstrating that resveratrol acts as a proteasome inhibitor, thereby suggesting a plausible mechanism by which resveratrol acts as an anti-inflammatory compound. We recently reported that increased NO levels, associated with ageing, may be attributable to increased degradation of IκB by ageing proteasomes, resulting in enhanced NF-κB activation [27]. We further demonstrated that these effects could be reversed by a variety of proteasome inhibitors [27].

Results of the current study are consistent with our previous studies of quercetin and δ-tocotrienol, which are common, naturally-occurring compounds that are commercially available as dietary supplements. We previously reported that proteasome inhibition by quercetin and δ-tocotrienol resulted in decreased proteolytic degradation of P-IκB which, in turn, decreased translocation of activated NF-κB to the nucleus, and depressed transcription of TNF-α and iNOS genes [5]. We also reported: 1) that quercetin and δ-tocotrienol inhibited secretion of TNF-α and NO production by LPS-stimulated murine macrophages *in vitro*[5]; 2) that macrophages from mice fed diets supplemented with these same compounds responded to LPS stimulation with decreased levels of mRNA for TNF-α and iNOS genes, and decreased secretion of TNF-α and NO production [23], and; 3) that serum TNF-α and NO levels were reduced in chickens fed diets supplemented with quercetin, riboflavin, or δ-tocotrienol [6]. In view of evidence linking dysregulated inflammatory responses to a variety of age-associated diseases (e.g. cancer, cardiovascular disease, dementia), findings that diet supplementation with quercetin, riboflavin, or δ-tocotrienol suppressed inflammatory responses in mice and chickens [6,27], raised the prospect that diet supplementation with comparable agents could have beneficial health effects in ageing humans [28].

Resveratrol and pterostilbene are present in grapes, blueberries, and red wine, and have been implicated as contributing factors to the lower incidence of cardiovascular disease in the French population despite their relatively high dietary fat intake [8-10]. Morin hydrate, which is an isoflavanoid found in tea leaves, has been shown to be an effective hypocholesterolemic agent [12]. In view of our findings described in the previous paragraph, we wanted to determine whether resveratrol, pterostilbene, and morin hydrate would also inhibit proteasome enzymatic activity and have anti-inflammatory effects comparable to quercetin and δ-tocotrienol. Our results indicated that resveratrol and pterostilbene were particularly potent proteasome inhibitors and anti-inflammatory agents, producing levels of inhibition that were at least comparable to, and often exceeding, that of the positive control (quercetin). Consequently, it appears likely that the beneficial nutritional affects of resveratrol and pterostilbene are due, at least in part, to their ability to inhibit NF-κB activation by the proteasome, thereby suppressing activation of pro-inflammatory cytokines and iNOS genes, resulting in decreased production of TNF-α, IL-1β, IL-6, and NO, in response to inflammatory stimuli.

Our current mechanistic findings are consistent with a recent report indicating that resveratrol modulates the stimulatory effects of TLR3 and TLR4, but not TLR2 or TLR9, ligands by suppressing NF-κB activation and cyclooxygenase-2 expression [29]. The suppressive effect of resveratrol appeared to be dependent upon Toll/IL-1R domain-containing adapter inducing IFN-β (TRIF) signaling pathways, as suppression was abolished in TRIF, but not MyD88 deficient mouse cells [29]. Our previous report, that proteasomes regulate TLR4 dependent inflammatory responses by affecting the TRIF/TRAM pathway, are entirely consistent with these findings [23]. These collective results indicating that dietary phytochemicals, such as trans- resveratrol, can modulate TLR-derived signaling, inflammatory target gene expression, and inflammatory cytokine and NO production, strongly suggest that these compounds can alter susceptibility to microbial infection and chronic inflammatory diseases [29].

## Conclusions

*trans*-Resveratrol, pterostilbene and morin hydrate inhibit the proteasome’s protease enzymatic activity, as well as the capacity of macrophages to produce inflammatory cytokines and NO in response to LPS stimulation. All three compounds inhibited chymotrypsin-like, trypsin-like, and post-glutamase proteasome enzymatic activity of cultured RAW 264.7 macrophage like cells. All three compounds inhibited production of nitric oxide (NO) and secretion of TNF-α by LPS-stimulated RAW 264.7 cells, and also inhibited expression of TNF-α, IL-1β, IL-6, and iNOS genes by LPS-stimulated RAW 264.7 cells and LPS-stimulated peritoneal macrophages from both C57BL/6 and BALB/c mice. NF-κB activation in LPS-stimulated HEK293T cells was also inhibited by resveratrol, pterostilbene and morin hydrate, providing a likely mechanism by which these proteasome inhibitors suppress inflammatory responses. It appears likely that the beneficial nutritional effects of resveratrol, pterostilbene, and morin hydrate as anti-inflammatory and anti-carcinogenic agents are due, at least in part, to their ability to inhibit NF-κB activation by the proteasome, thereby suppressing activation of pro-inflammatory cytokines and iNOS genes, resulting in decreased production of TNF-α, IL-1β, IL6, and NO, in response to inflammatory stimuli.

## Abbreviations

HEK293T cells: human embryonic kidney 293 T cells; LPS: lipopolysaccharide; TNF-α: tumor necrosis factor-α; IL-1β, interleukin-1β; IL-6, interleukin-6; NO: nitric oxide; iNOS: inducible nitric oxide synthase; NF-κB: nuclear factor-kappa B; P-IκB: phosphorylated-inhibitor kappa B; 1. LPS (Control group): media + cells + LPS (10 ng/well) + 0.2 % or 0.4 % dimethyl sulfoxide (DMSO); 2. Resv: (Resveratrol); 3. Pter: (Pterostilbene); 4. Mori: (Morin hydrate); 5. Nico: Nico (Nicotinic acid); 6. Quer: (Quercetin).

## Competing interests

The authors declare that they have no competing interests.

## Authors’ contributions

All the authors were involved in the design of this study. Dr. XQG (Postdoctoral fellow M.D.) carried out TNF-α, NO, and gene expression assays. Ms. JCR carried out assays of chymotrypsin-like, trypsin-like, and post-glutamase activity. Ms. SJ carried out NF-κB assays in Dr. SNV laboratory. Dr. CJP edited the manuscript. All the authors have read and approved the final version.
